# Effect of replacing soybean meal (*Glycine max*) with sesame meal (*Sesamum indicum*) on productive traits, carcass characteristics, and gross profit margin in fattening lamb’s diets

**DOI:** 10.1007/s11250-022-03406-1

**Published:** 2022-11-28

**Authors:** Eleazar Pérez-Trejo, Héctor M. Andrade-Montemayor, Lizbeth E. Robles-Jimenez, María Concepción Méndez Gómez Humarán, Elba Orozco-Estrada, Jesús Hernández-Hernandez, Einar Vargas-Bello-Pérez, Manuel Gonzalez-Ronquillo

**Affiliations:** 1 Licenciatura en Medicina Veterinaria Y Zootecnia, Facultad de Ciencias Naturales, Universidad Autónoma de Querétaro, Av. De Las Ciencias S/N Col. Juriquilla, Santiago de Querétaro, CP 76530 Querétaro, México; 2 Universidad Autónoma del Estado de México, Facultad de Medicina Veterinaria Y Zootecnia, Instituto Literario 100 Ote., C.P. 50000, Toluca, Estado de México México; 3 Department of Animal Sciences, School of Agriculture, Policy and Development, University of Reading, Earley Gate, P.O. Box 237, Reading, RG6 6EU UK

**Keywords:** Lambs, Protein sources, Sesame meal, Soybean meal, Profitability

## Abstract

The objective of this study was to determine the effect of replacing soybean meal (*Glycine max*) with sesame meal (*Sesamum indicum*) on productive traits, carcass characteristics, and gross profit margin (GMP) in fattening lamb’s diets. For this, 42 Katahdin lambs were divided into three treatments in duplicate: basal diet + soybean meal (100S), basal diet + sesame meal/soybean meal (50/50SA), and basal diet + sesame meal (100A). Dry matter intake, daily weight gain (DWG), total kg gained (KgT), feed conversion (FC), and feed efficiency (FE) were evaluated; upon reaching the weight for sale, the animals were slaughtered, and hot carcass weight (HCW) was evaluated. The results were analyzed with a completely randomized design with repeated measures. Regarding time, no differences were found between treatments, for DWG (0.171 ± 0.006 kg/d), FC (6.7 ± 0.55), FE (0.175 ± 0.02), KgT (2.86 ± 0.13 kg), HCW (50.97 ± 0.79 kg), as well as for chest depth (26.96 ± 0.33 cm), leg width (20.63 ± 0.028 cm), leg diameter (60.7 ± 0.44 cm), and ribs width (24.05 ± 0.14 cm). GPM was 16.50%, 18.63%, and 19.97% for 100S, 50/50SA, and 100A, respectively. Overall, in fatting lamb diets, replacing soybean meal with sesame meal by either 50% or 100% substitution could be a feasible feeding strategy as in both cases, gross profit was increased, and no negative effects were found for productive traits and carcass quality.

## Introduction

Mexican sheep farming is mostly devoted to Barbacoa market, which is a traditional Mexican dish (Partida de La Peña et al., 2013). Although this market can accept any type of sheep regardless of age, sex, breed, or production system, animals that reach the best prices are male sheep under 1 year of age fattened with diets based on concentrated feeds (Partida de La Peña et al. 2013).

In these systems, lambs are fattened in feedlots with diets rich in concentrates and have high daily weight gains (DWG) and meat tenderness as they are slaughtered at early ages (Shirima et al. 2014). In this production system, there is a great dependence on grains (corn and sorghum) and oilseed by-products (soybean meal) together with corn stovers, oat hay, and/or alfalfa hay. Feed conversion (FC) is important since grain prices have historically been on the rise (CEDRSSA 2021), while the livestock prices (live animals) have gone down (OCDE/FAO 2017). This result in a negative combination, leading to a considerable reduction in producers’ profits and in some cases even leading to have losses; thus, it is important to find alternative feedstuffs to reduce production costs.

For fattened lambs to have a DWG higher than 200 g/day, dietary crude protein (CP) contents between 13 and 17% and dietary energy of 2.4 Mcal ME/kg DM must be included (NRC 2007). In Central Mexico, one alternative to reach this productive goal and satisfy these nutrients requirements is the use of protein feedstuffs such as soybean meal (Shen et al. 2018). However, due to soybean meal price, sesame meal (*Sesamum indicum*) could be an alternative. This is a by-product from the sesame oil industry and is characterized by having protein contents of 40% CP and an energy level of 2.98 Mcal ME/kg DM (Hassan et al. 2013).

Due to the aforementioned background, the objective of the present study was to evaluate the effect of the replacing of soybean meal with sesame meal in fattening lamb diets, on productive traits, carcass characteristics, and gross profit margin (GPM).

## Materials and methods

The study was carried out at the farm “Agropecuaria el Fénix,” in Cadereyta de Montes, Mexico. This farm is located at 3240 m above sea level. The predominant climate in this region is temperate semi-dry with an average rainfall of 480 mm and an average annual temperature of 16° C (INAFED 2021). All experimental procedures were approved by the Bioethics Committee of the Autonomous University of the State of Mexico (UAEMex4974 / 2020).

### Animals and diets

Forty-two 4-month-old Katahdin lambs (27 ± 3 kg), not castrated, were randomly selected and divided into 6 groups, in individual roofed pens (3 × 2 m) with raised (50-cm height) slatted floors with automatic drinkers and feed bunks.

Animals were grouped in a completely randomized design with 3 treatments (two repetitions per treatment). For 84 days, animals received a basal diet that was formulated based on ground corn grain, waste biscuit, grass hay, a premix of minerals and vitamins, calcium carbonate, and sesame meal (100S) (*n* = 16) or soybean meal (100SB) (*n* = 13) and their 50/50 combination (50SSB) (*n* = 15).

Diets were isonitrogenous (15.5% CP/kg DM) and isoenergetic (13.3 MJ, ME/kg DM) (Table 1). Rumen degradable protein (RDP, g/kg) and EM (MJ/Kg DM) were calculated according to NRC (2007). Animals had 10 days for diet adaptation. Dry matter intake was recorded daily, and body weights were registered every 12 days to calculate DWG and FC.

**Table 1:** Ingredients and chemical composition from lamb diets containing sesame meal (100S) or soybean meal (100SB) or their 50/50 combination (50SSB)

	Soybean meal (100SB)	50SB/50S (50SSB)	Sesame meal (100S)
Ingredients (g/kg)			
Grounded corn grain	480	460	440
Waste biscuit	300	300	300
Sesame meal	0	90	180
Soybean meal	140	70	0
Grass hay	50	50	50
Vitamin and mineral premix	20	20	20
Calcium carbonate	10	10	10
Chemical composition (g/kg)			
Dry matter	885	883	881
Organic matter	934	937	940
Crude protein	159	156	153
Rumen degradable protein	114	116	118
Ether extract	19.1	15.0	11.3
Neutral detergent fiber	128	130	149
Acid detergent fiber	73.0	78.2	83.7
Lignin	4.1	4.6	5.6
Ash	65.9	62.1	59.9
Metabolizable energy (MJ/kg DM)	13.2	13.3	13.4

Contained per kg of diet: 16% calcium, 1.2% phosphorus, 2% sulfur, 17.00 mg/kg selenium, 2000 mg/kg zinc, 160,000.00 IU/g vitamin A

The study was made up of 6 stages of 14 days each, for which 10 days were provided for the adjustment of the diet and 4 days later to obtain feces, urine, and ruminal fluid. The feces were collected in their entirety, preserving them in hermetic bags, to subsequently form a single sample which was dried at 55 °C, for 48 h, and then crushed in a Willey mill with a 1-mm sieve for further analysis. For urine, a sample of 100 ml per day was taken. The ruminal fluid was obtained by probing, and 50 ml were obtained per day at the end of each experimental period.

Every week, diet samples were taken. These samples were ground using a 1-mm Wiley mill screen (Arthur H. Thomas, Philadelphia, PA) and analyzed later for total N (954.01) (AOAC 2005), dry matter (DM), ash, and organic matter (OM) (AOAC 2005). Dietary fiber fractions were analyzed for neutral detergent fiber (NDF), acid detergent fiber (ADF), and lignin, using the methods described by Van Soest et al. (1991). Total fat was done using standard procedure from (920.39) AOAC (AOAC 2005).

### Carcass evaluation

After 84 days, lambs were slaughtered, and hot carcass weight (HCW) and hot carcass performance were determined. Back fat depth, leg length and diameter, chest depth, and carcass length were measured.

### Profitability of lamb fattening

Gross profit margin (GPM) and running costs (RC) were evaluated. For GPM, gross profit (income–expenses) was divided by total income (lamb sales and manure sales), and this was multiplied by 100. Running costs were determined by dividing the expenses (purchase of animals, feed, labor, veterinary supplies, and energy costs for fattening) by the total income, and this was multiplied by 100 (BCcampus 2021).

### Statistical analysis

Data on live weight, DWG, and total gain were analyzed using a completely randomized design with repeated measures over time. Data was analyzed using analysis of variance with the SAS program (2002), according to the following model.

$$\mathrm{Yijk}=\mathrm\mu+\mathrm{period}\;\mathrm i+\mathrm{treatment}\;\mathrm j+(\mathrm{period\;x}\;\mathrm{treatment})\mathrm{ij}+\mathrm{eij}$$

where Yijk = live weight, DWG, and total gain, μ = general mean, periodi = effect of the period (*n* = 5), treatmentj = effect of the treatment (*n* = 3), the interaction (period × treatment) ij = effect of the period by the treatment, and eijk = random error.

The final live weight and carcass yield data were analyzed using a completely randomized design with the SAS (2002) program according to the model:

$$\mathrm{Yj}=\mathrm\mu+\mathrm{treatmentj}+\mathrm{ej}$$

where Yj = final body weight and carcass yield, μ = general mean, treatment j = treatment effect (*n* = 3), and ej = random error. The means of each variable (*P* < 0.05) were compared using Tukey test.

## Results

Productive traits from lambs fed on experimental diets are shown in Table 2. No differences were observed between treatments on daily weight gain (0.171 ± 0.006, kg/day), feed conversion (6.73 ± 0.55), feed efficiency (0.175 ± 0.02), and dry matter intake (1.269 ± 0.09 kg/day). The last period had the lowest (*P* < 0.05) DWG and DMI. Daily weight gains were higher on period 1.

**Table 2:** Productive traits from lamb diets containing sesame meal (100S) or soybean meal (100SB) or their 50/50 combination (50SSB)

Parameters	Treatment (T)		Period (P)								*P*-value	
	100SB	50SSB	100S	1	2	3	4	5	SEM	T	P	T × P
Initial weight	39.5	36.7	37.9	31.0^c^	35.5^bc^	40.4^abc^	45.6^ab^	49.7^a^	2.15	0.654	0.001	0.946
kg/period	2.68	2.96	2.96	3.60	3.62	3.62	3.82	2.90	0.28	0.716	0.345	0.965
DWG (kg)	0.16	0.17	0.17	0.23^d^	0.20^c^	0.20^a^	0.21^a^	0.17^b^	0.01	0.774	0.001	0.945
DMI (kg/d)	1.38	1.27	1.21	1.15^ba^	1.41^a^	1.46^a^	1.41^a^	1.00^b^	0.05	0.128	0.002	0.668
FC	7.5	6.2	6.5	5.0	7.2	8.2	7.1	6.3	0.84	0.547	0.350	0.995
FE	0.14	0.19	0.19	0.23	0.15	0.14	0.15	0.17	0.02	0.365	0.3834	0.997

a–c Means with the same letter are not significantly different at *P* < 0.05. *SEM* standard error of the mean, *DWG* daily weight gain, *DMI* dry matter intake, *FC* feed conversion, *FE* feed efficiency

The best gross profitability (Table 3) was obtained from 100S (19.97%), followed by 50SSB (18.63%), and the lowest was 100SB (16.50%).

**Table 3:** Economic analysis from fattening lambs offered diets containing sesame meal (100S) or soybean meal (100SB) or their 50/50 combination

	Treatment		
Variable	100SB	50SSB	100S
Total income	$1,639.49	$1,770.49	$1,885.78
Total expenses	$1,368.91	$1,440.70	$1,509.24
Gross profit	$270.58	$329.78	$376.54
Gross profit margin	16.50%	18.63%	19.97%
Cost operation margin	83.50%	81.37%	80.03%

^*^This analysis considers a scenario where no mortality was present in any of the groups of lambs

Data is based on an exchange rate of $19.53 Mexican pesos per US dollar

The animals with the highest finishing weight (Table 4) were those of the soybean meal group, and the 50/50 group had the lowest weight. However, the 50/50 group had the lightest carcasses, compared to soybean meal and sesame meal. Hot carcass weight was similar between treatments (50.97 ± 0.79). Chest depth, leg width, leg diameter, and rib width were similar between treatments. Carcass length was higher with soybean meal compared with the other diets*.*

**Table 4:** Carcass yield from fattening lambs offered diets containing sesame meal (100S) or soybean meal (100SB) or their 50/50 combination (50SSB)

Parameter	Treatment			SEM	*P*-value
	100SB	50SSB	100S		
Final weight, kg	51.9^a^	46.0^b^	49.8^ab^	1.33	0.024
Hot carcass weight, kg	26.0^a^	23.2^b^	25.9^a^	0.76	0.014
Carcass yield, %	50.2	50.6	52.0	0.67	0.155
Carcass length, cm	70.6^a^	68.5^b^	68.3^b^	0.37	0.001
Chest depth, cm	26.9	27.3	26.5	0.29	0.107
Leg width, cm	20.6	20.6	20.6	0.31	0.984
Leg diameter, cm	61.3	60.3	60.4	0.62	0.554
Rib width, cm	24.1	24.1	23.8	0.52	0.893

^abc^ Means with the same letter are not significantly different at *P* < 0.05. *SEM* standard error of the mean

## Discussion

In lamb fattening systems, one of the main ways to improve farmer’s profit is to reduce feeding cost or improve feed efficiency. Therefore, this objective can be achieved by improving weight gains and feed conversion (De Sousa et al. 2012). One strategy could be by replacing expensive feedstuffs with alternative resources. In this sense, in this study, soybean meal was replaced by sesame meal, which is normally less expensive than soybean meal in central Mexico.

According to the present study, lamb productive parameters were not affected after replacing soybean meal with sesame meal. This agrees with Mahmoud and Bendary (2014), who fed sesame meal on fattening lamb at an inclusion of 12.5% DM with 12.5% DM Nigella sativa meal to replace ingredients rich in protein such as soybean meal and cottonseed, and inclusion of sesame meal led to increase profitability.

Similarly, with fattening Awassi lambs, Obeidat et al. (2009) replaced soybean meal (16% of the total diet) either partially (8% soybean meal + 8% sesame meal) or totally (0% soybean meal + 16% sesame meal) with sesame meal and reported lower DMI and DWG compared to soybean meal fed animals. However, in the present study, no differences were detected for DMI. It is noteworthy of mentioning that in Obeidat et al. (2009) study, feed conversion and the cost per kg of lamb were improved in those animals feed only on sesame meal. This coincides with the fact that sesame meal could be a more profitable feeding strategy compared to the use of soybean meal.

Gebreslassie and Melaku (2009) found a positive effect when replacing wheat bran with sesame meal in lambs fed on a stubble diet. Treatments in that study consisted of supplementing 300 g/day of a supplement only based 100% sesame meal, 100% wheat bran, 65% wheat bran + 35% sesame meal, or 35% wheat bran + 65% sesame meal. Daily weight gains and feed conversion were higher in animals receiving the highest content of sesame meal. It is important to mention that this sesame diet had the highest protein content than that of wheat bran. This study also supports our findings showing that sesame meal (153 g/kg CP) can be used to replace soybean meal (159 g/kg CP) in lamb diets.

In another study, Hassam et al. (2013) replaced wheat bran and peanut meal with sesame meal, at 15% or 20% inclusion, where at 15% inclusion level, daily weight gains (0.143 kg) were lowest, followed by inclusion at 20% (0.166 kg) being highest in the diet without sesame meal (0.179 kg). For feed conversion, the highest value was for 15% sesame meal (9.02) followed by 20% sesame meal (7.65) and the lowest for the diet without sesame meal (6.87). These results differ from those of our study probably due to the difference in the quantity of inclusion of sesame meal in the diets, which in our study was 50 and 100% compared to 15 and 20% in the study mentioned above.

In this study, the highest GPM was obtained from animals fed with 100% sesame meal. This is because the sesame meal price is considerably lower than that of soybean meal (sesame meal is 0.16 USD, while soybean meal is 0.41 USD). This calculation was based on the existing prices during the study. Our results coincide with studies using Awassi lambs (Obeidat et al. 2009; Abo Omar 2002). In Obeidat et al. (2009) study, lambs had lower DWG and higher FC when fed with 100% soybean meal, which is a clear indicator that the differential in the price of sesame meal is significant. On the contrary, Abo Omar (2002) reported that inclusion of sesame meal did improve the DWG and FC with regard to diets without sesame meal. In this study, there was no significant difference in productive parameters; however, production costs were lower with sesame meal inclusion.

In this study, except for carcass length, carcass quality characteristics were similar between treatments. Fitwi and Tadesse (2013) found an improvement in the performance of Koraro sheep carcasses with diets based on teff stubble and with different inclusion levels of sesame meal instead of wheat bran. In that study, carcass yield was similar between different inclusion levels of sesame meal, and this could be explained because diets were isoproteic and isoenergetic. Similarly, Gorbani et al. (2018) used Zel lambs and reported no effects on carcass characteristics when replacing soybean meal with sesame meal at different levels of inclusion (0%, 25%, 50%, 75%, and 100%), and diets were also isoproteic and isoenergetic.

For lamb production, the search for protein-feed alternatives that are less expensive compared to the conventional use of soybean meal is important. Especially when the use of alternative sources such as sesame meal does not have detrimental effects on productive traits and carcass characteristics. This suggests that the diets with sesame meal will have a lower price without reducing productivity and carcass quality, resulting in a lower production cost and a higher profit margin*.*

## Conclusions

In fattening lamb diets, replacing soybean meal with sesame meal by either 50 or 100% substitution could be a feasible feeding strategy as in both cases, gross profit was increased, and no negative effects were found for productive traits and carcass quality.

## Data Availability

Data will be made available on reasonable request.
